# Nonlinear Generation of Electromagnetic Waves through Induced Scattering by Thermal Plasma

**DOI:** 10.1038/srep17852

**Published:** 2015-12-09

**Authors:** E. M. Tejero, C. Crabtree, D. D. Blackwell, W. E. Amatucci, M. Mithaiwala, G. Ganguli, L. Rudakov

**Affiliations:** 1 Plasma Physics Division, Naval Research Laboratory, Washington, DC 20375; 2 Icarus Research Inc., P.O. Box 30780, Bethesda, MD 20824.

## Abstract

We demonstrate the conversion of electrostatic pump waves into electromagnetic waves through nonlinear induced scattering by thermal particles in a laboratory plasma. Electrostatic waves in the whistler branch are launched that propagate near the resonance cone. When the amplitude exceeds a threshold ~5 × 10^−6^ times the background magnetic field, wave power is scattered below the pump frequency with wave normal angles (~59°), where the scattered wavelength reaches the limits of the plasma column. The scattered wave has a perpendicular wavelength that is an order of magnitude larger than the pump wave and longer than the electron skin depth. The amplitude threshold, scattered frequency spectrum, and scattered wave normal angles are in good agreement with theory. The results may affect the analysis and interpretation of space observations and lead to a comprehensive understanding of the nature of the Earth’s plasma environment.

Induced nonlinear scattering is fundamental to weak turbulence and impacts plasma evolution when wave amplitudes are above a nonlinear threshold. It is particularly important to the dynamics of space plasmas. This kinetic nonlinear wave-particle interaction has been shown to lead to a magnetospheric cavity^1^ that can amplify whistler waves during active periods^2^ and regulate the trapped electron population in the radiation belts. It can also influence the evolution of solar wind turbulence^3^, and explain the longevity of electron beams in the solar wind^4^.

Generalization of the electrostatic theory of induced nonlinear scattering of lower hybrid waves^5^ to include the electromagnetic effects^6^ showed that electrostatic waves in the intermediate frequency range, Ω_*i*_ < *ω* < Ω_*e*_ (Ω_*i*,*e*_ are the ion and electron gyrofrequencies), with a large wave normal angle may be scattered by a resonant nonlinear interaction with thermal electrons into electromagnetic waves with smaller wave normal angles^6^,^7^. This Report describes the first laboratory experiment that demonstrates the nonlinear conversion process.

This conversion process forms the theoretical basis^1^ for producing electromagnetic energy in the ionosphere by seeding weak turbulence of lower hybrid waves and scattering them into whistlers. The whistlers with large group velocity can propagate out of the ionosphere and transport energy over an extended volume to affect the plasma dynamics at remote locations. This suggests the possibility of active radiation belt remediation^1^,^7^,^8^. Since nonlinear scattering is key to a number of important plasma processes, the primary objective of our experiment is to isolate and validate this mechanism in a controlled and repeatable laboratory experiment.

The observed effect that will be discussed in this Report is determined to be nonlinear induced scattering by particles on the basis of three key features. (1) Given sufficient amplitude of a pump wave with frequency *f*_*pump*_ and 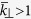, a new spectral feature, the scattered wave, is observed at a downshifted frequency *f*_*pump*_ − Δ*f* and 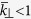, while no corresponding low frequency wave is seen at the frequency Δ*f*, where the normalized perpendicular wave vector 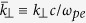, *ω*_*pe*_ is the electron plasma frequency, and *c* is the speed of light. (2) The amplitude of the scattered wave exhibits a quadratic dependence on the pump wave amplitude above a threshold, below which no scattered wave is observed. (3) The beat wave that would be present between the pump and scattered waves is in Landau resonance with the background thermal electrons 2*π*Δ*f*/(*v*_*te*_Δ*k*_*z*_) ~ 1, where *v*_*te*_ is the electron thermal speed and Δ*k*_*z*_ is the absolute value of the difference between the parallel wave vectors of the pump and scattered waves. Furthermore, the perpendicular wavelength of the scattered waves is an order of magnitude larger than that of the pump wave and longer than the electron skin depth.

The experiments were conducted in the Space Physics Simulation Chamber (SPSC) at the Naval Research Laboratory (NRL)^9^,^10^ in the main chamber section, which is a 5-m long, 2-m diameter cylindrical vacuum chamber with a large area hot filament plasma source. The operational parameters for the steady-state argon plasma are plasma density *n* = 3 × 10^10^ cm^−3^, ion and electron temperatures *T*_*i*_ ≈ 0.05 eV and *T*_*e*_ = 1.3 eV, respectively and uniform axial magnetic field *B* = 5 mT. The electron-neutral collision frequency is *ν*_*en*_ ≈ 10^6^ s^−1^ for a neutral density of *n*_*n*_ ~ 10^13^ cm^−3^. These parameters correspond to scaled conditions of low-*β* (~6 × 10^−4^) and dense (*ω*_*pe*_/Ω_*e*_ ~ 11) plasma. The effective plasma column diameter and length over which the plasma is observed to be uniform are 1 m and 5 m, respectively.

The plasma measurements were made with probes that are scanned in a two-dimensional plane, 2 m along the axis of the chamber and 60 cm in one direction perpendicular to the background field. A guarded Langmuir probe was used to measure density and electron temperature. Three-axis magnetic field probes were used to measure the wave fields. The pump wave was launched using a 1.2-m-long linear antenna aligned axially and positioned at the radial edge of the plasma column. The antenna consists of four 30 cm × 30 cm panels that can be independently phased. For all of the experiments presented, the first and third panels and the second and fourth panels were electrically connected, while the two pairs of panels were driven 180° out of phase. The scattered waves are observed for a range of pump frequencies. See Fig. 1 for a schematic of the experimental setup.

Figure 2 depicts typical spectra for an experimental run where the pump wave amplitude is above (solid black) and just below (dashed red) the nonlinear scattering threshold. They were measured approximately 15 cm from the antenna. The magnitude of the measured magnetic field is plotted as a function of frequency. The pump wave at *f*_*pump*_ = 10 MHz is truncated in the large amplitude case with peak at 159 nT (*δB*/*B* ≈ 3 × 10^−5^). The spectral feature peaking at *f* ~ *f*_*pump*_ − Δ*f* (Δ*f* = 40 kHz) is the induced scattered signal. The pump wave amplitude decreases by about an order of magnitude outside the resonance cone, but it is measureable over the plasma column. The peak amplitude of the scattered wave occurs far from the antenna near the center of the plasma column, not where the pump wave amplitude peaks. The relatively broadband continuous nature of the scattered signal is due to the ease of satisfying the Landau resonance condition of the beat waves. There is no corresponding low frequency spectral feature at *f* ~ Δ*f* indicating that this is not a three-wave process.

If the sideband at 9.96 MHz were due to a three-wave decay process, we can estimate the expected magnitude of the wave magnetic field of the low frequency (40 kHz) wave, by using the fact that the plasmon density *N* = *W*/*ω* of the two daughter waves in parametric three-wave decay should be equal. We use the cold plasma dispersion relation to estimate the wave energy density *W* for the sideband and low frequency wave. We estimated the expected magnitude of the low frequency wave to be 3 nT, which would be measureable above the noise floor of approximately 0.5 nT.

The plot in Fig. 3 shows the normalized peak scattered wave amplitude as a function of normalized pump wave amplitude for RF voltages from 0.07–1.0 V applied to the antenna. The plot demonstrates the nonlinear threshold for the scattering with a value of *δB*/*B* = 5 × 10^−6^. These observations confirm the theoretical predictions^1^ that a large amplitude is not required to initiate nonlinear induced scattering as long as the scattering rate exceeds the linear damping rate. The plot also indicates a nonlinear dependence on the normalized pump wave amplitude and shows the corresponding parabolic best fit (dashed line). Applying a linear fit results in a mean squared error that is an order of magnitude larger than that from the parabolic fit. Since 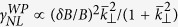
7, this confirms that the scattered waves are due to a nonlinear wave-particle mechanism, as a linear dependence 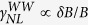 would follow from three-wave parametric decay^11^.

The cross-correlation between a stationary reference probe and a probe moved within the experimental volume is used to map the spatial variation of the magnitude and phase of the launched and scattered waves. Spatial Fourier transforms of this data yield the **k**-spectrum for these waves. A summary of these results is shown in Fig. 4, where the normalized power spectrum is plotted as a function of *k*_⊥_ (top pane) and *k*_||_ (bottom pane) both normalized to the electron skin depth *c*/*ω*_*pe*_. The dominant wave mode for the pump wave (dashed red line) exhibited a *k*_||_ = −7 ± 2 m^−1^ and *k*_⊥_ = 82 ± 14 m^−^1, results in a wave normal angle *θ* ~ 85^°^. The wave normal angle is defined as the angle between **k** and the magnetic field. The spatial Fourier transform indicates 1–2 dominant modes, which allows us to fit to the phase data to achieve better wave vector resolution than the total travel would initially indicate. The perpendicular wave vector normalized to the electron skin depth 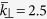 demonstrates that the launched wave is predominantly electrostatic.

Cross-correlation measurements for the scattered waves were obtained by averaging an increased number of shots: 100,000 shots were used to remove the random nature present in the waves. Figure 4 shows the large change in perpendicular wave vector that is observed between the pump and scattered waves. The pump wave at 10 MHz with *k*_⊥_ = 82 m^−1^ is scattered to waves with *k*_⊥_ = 10 m^−1^. The dominant scattered wave was observed to have *k*_*z*_ = ±6 m^−1^, resulting in a wave normal angle *θ* ~ 59^°^. The intermediate frequency cold plasma dispersion relation for the experimental parameters is 
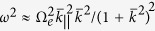
. The shaded region in Fig. 4 indicates where the waves are predominantly electrostatic 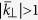 with a dispersion of 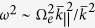 (i.e. electrostatic whistlers); the pump 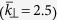 scatters into electromagnetic waves 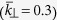 with dispersion 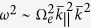 (i.e. oblique electromagnetic whistlers).

Using the measured *k*_||_ values of the pump and scattered waves, we find that Δ*ω*/Δ*k*_*z*_ ~ *v*_*te*_/2, which is consistent with the condition that the beat wave be in Landau resonance with the electrons. We also use the nonlinear growth rate, Eq. (11) from Ganguli *et al.*^6^, to obtain the fluctuating magnetic fields and predict the expected frequency spectrum of scattered waves. To generate this spectrum the calculated scattered fields were integrated over all wave propagation angles and plotted as a function of frequency. The resulting spectrum is overlaid (dotted blue line) on Fig. 2. The predicted peak frequency of the scattered waves ~9.963 MHz agrees favorably with the observed downshift of Δ*f* ~ 40 kHz.

In a theoretical study of nonlinear parametric processes, Tripathi *et al.*^12^ showed that the dominant channel of decay for a coherent, large amplitude lower hybrid wave is through the three-wave resonant decay to an ion acoustic and another lower hybrid wave when *T*_*e*_/(2*T*_*i*_) > 4 and through nonlinear induced scattering otherwise. When *T*_*e*_ ~ *T*_*i*_, as is the case for many space plasmas^6^, the low-frequency wave is damped due to ion Landau damping, and thus nonlinear scattering (quasi-mode decay) is the dominant nonlinear decay channel. In the SPSC experiments *T*_*e*_ ≫ *T*_*i*_ and thus Landau damping is negligible, however, collisional dissipation is large for the low-frequency (40 kHz) waves (collisional dissipation was not considered in Tripathi *et al.*), which makes the nonlinear scattering the preferred nonlinear decay channel^13^ over the three-wave decay through ion-acoustic waves for our experiments.

The experiments discussed above demonstrate that nonlinear scattering by thermal particles can convert an electrostatic wave in the intermediate frequency range in a magnetized plasma to an electromagnetic wave. This can significantly affect the saturation and evolution of three-dimensional weak turbulence in the intermediate frequency range. In previous experiments^14^, the analysis was restricted to the electrostatic formulation. Nonlinear scatterings constitute weak turbulence that determines the evolution of plasmas. The geometrical constraints in a laboratory device make it difficult to replicate fully developed weak turbulence, which requires an ensemble of plane waves with random phases. Therefore, we isolated nonlinear scatterings, which are fundamental to turbulence and can be demonstrated with narrowband waves in a laboratory device. This allowed detailed characterization of this important plasma micro-process and validation of the theory. The theory, validated in low-*β* plasma, can be extended to higher *β* plasmas, e.g. solar wind plasmas, which are not easily accessible in the laboratory. Induced scattering by thermal particles may also be important in the solar wind especially when scale sizes become comparable to the ion skin depth or smaller^3^. The wave-particle scattering is less sensitive to the spatial extent of the experimental device or to the box size in a numerical simulation, because the momentum conservation in this case involves the generalized momentum of the background plasma. Consequently, the scattered wave vectors can adjust to the geometrical constraints of the experimental device or the box size in a numerical simulation. In a finite volume or inhomogeneous system the quantization effects of scattered wave vectors can be easily accommodated in wave-particle scattering but not in wave-wave scattering processes such as decay or coalescence.

## Additional Information

**How to cite this article**: Tejero, E. M. *et al.* Nonlinear Generation of Electromagnetic Waves through Induced Scattering by Thermal Plasma. *Sci. Rep.* **5**, 17852; doi: 10.1038/srep17852 (2015).

## Figures and Tables

**Figure 1:**
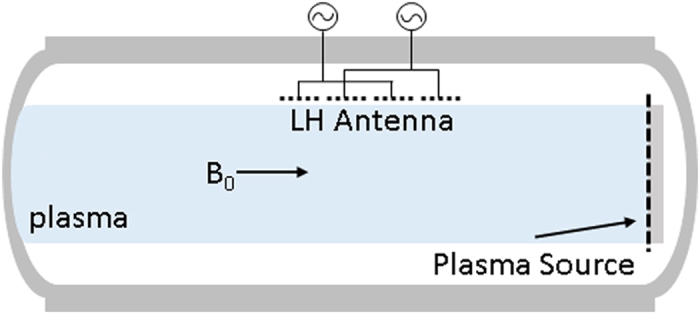
Schematic of the NRL Space Physics Simulation Chamber depicting the experimental setup.

**Figure 2:**
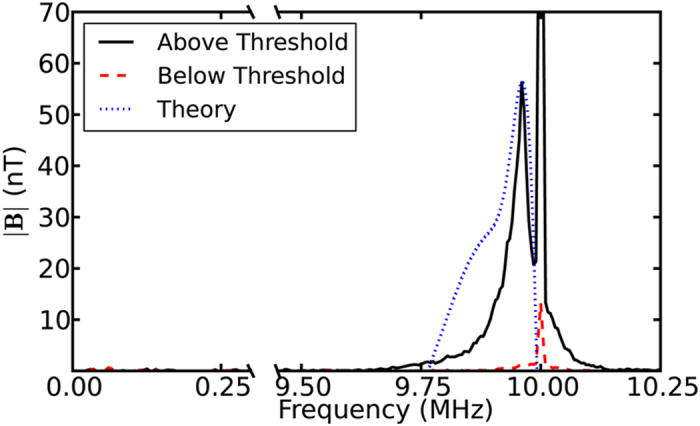
Typical spectra showing the magnitude of B as a function of frequency with the normalized theoretically predicted spectrum for the scattered waves (dotted blue).

**Figure 3:**
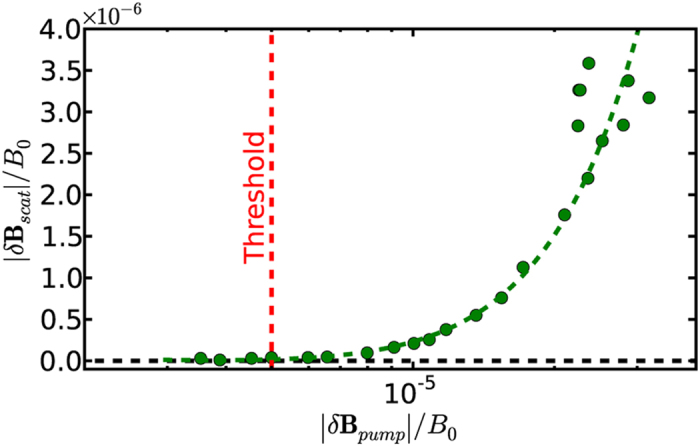
Scattered wave magnetic field amplitude at the peak frequency versus pump wave amplitude.

**Figure 4:**
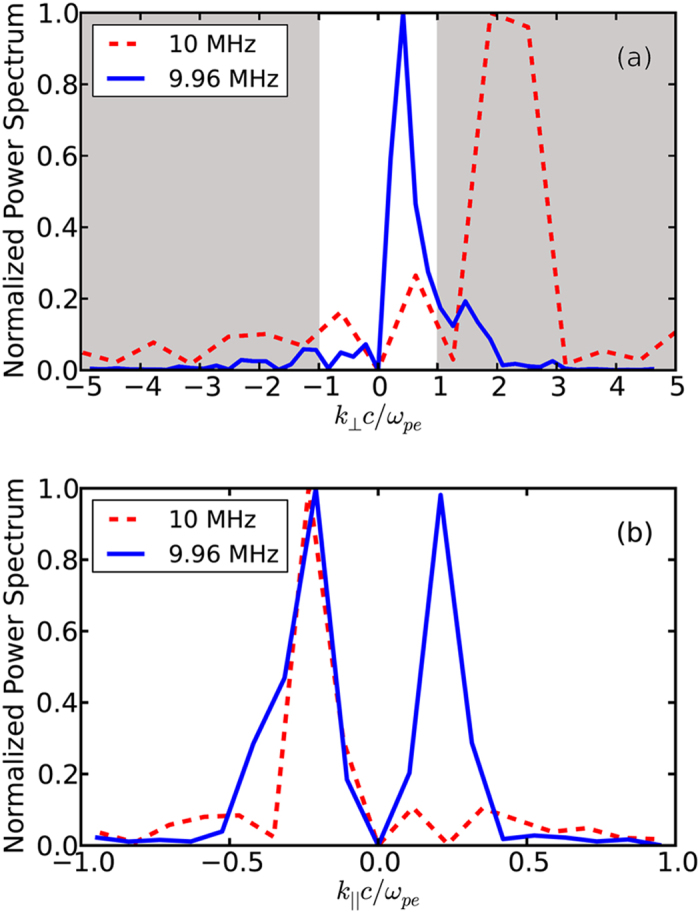
Comparison of the perpendicular (top) and parallel (bottom) wavelengths between the pump wave at 10 MHz and the scattered waves at 9.96 MHz. The shaded region indicates where the waves are predominantly electrostatic.

## References

[b1] Crabtree C. *et al.* Weak turbulence in the magnetosphere: Formation of whistler wave cavity by nonlinear scattering. Phys. Plasmas 19, 032903 (2012).

[b2] Ganguli G., Rudakov L., Crabtree C. & Mithaiwala M. Multi-pass whistler gain in a magnetospheric cavity due to induced nonlinear scattering. Geophys. Res. Lett. 39, L16105 (2012).

[b3] Rudakov L., Mithaiwala M., Ganguli G. & Crabtree C. Linear and nonlinear landau resonance of kinetic alfven waves: Consequences for electron distribution and wave spectrum in the solar wind. Phys. Plasmas 18, 012307 (2011).

[b4] Thejappa G. & MacDowall R. J., Bergamo, M. & Papadopoulos, K. Evidence for the oscillating two stream instability and spatial collapse of langmuir waves in solar type iii radio burst. Astrophys. J. Lett. 747, L1 (2012).

[b5] Hasegawa A. & Chen L. Theory of plasma heating by nonlinear excitation of lower hybrid resonance. Phys. Fluids 18, 1321 (1975).

[b6] Ganguli G., Rudakov L., Scales W., Wang J. & Mithaiwala M. Weak turbulence in the magnetosphere: Formation of whistler wave cavity by nonlinear scattering. Phys. Plasmas 17, 052310 (2010).

[b7] Mithaiwala M., Rudakov L., Ganguli G. & Crabtree C. Stability of an ion-ring distribution in a multi-ion component plasma. Phys. Plasmas 17, 042113 (2010).

[b8] Ganguli G., Rudakov L., Mithaiwala M. & Papadopoulos K. Generation and evolution of intense ion cyclotron turbulence by artificial plasma cloud in the magnetosphere. J. Geophys. Res. 112, A06231 (2007).

[b9] Blackwell D. D., Walker D. N. & Amatucci W. E. Whistler wave propagation in the antenna near and far fields in the naval research laboratory space physics simulation chamber. Phys. Plasmas 17, 012901 (2010).

[b10] Amatucci W. E. *et al.* Whistler wave resonances in laboratory plasma. IEEE Trans. Plasma Sci. 39, 637 (2011).

[b11] Liu C. S. & Tripathi V. K. Parametric instabilities in a magnetized plasma. Phys. Rep. 130, 143 (1986).

[b12] Tripathi V. K., Grebogi C. & Liu C. S. Unified formalism of lower hybrid parametric instabilities in plasmas. Phys. Fluids 20, 1525 (1977).

[b13] Porkolab M. Theory of parametric instability near the lower hybrid frequency. Phys. Fluids 17, 1432 (1974).

[b14] Porkolab M. & Chang R. P. H. Nonlinear wave effects in laboratory plasmas: A comparison between theory and experiment. Rev. Mod. Phys 50, 745 (1978).

